# Thermodynamic Cyclic Voltammograms Based on *Ab Initio* Calculations: Ag(111) in Halide-Containing Solutions

**DOI:** 10.1021/acs.jctc.0c01166

**Published:** 2021-02-19

**Authors:** Nicolas G. Hörmann, Karsten Reuter

**Affiliations:** † Chair of Theoretical Chemistry and Catalysis Research Center, Technische Universität München, Lichtenbergstr. 4, 85748 Garching, Germany; ‡ Fritz-Haber-Institut der Max-Planck-Gesellschaft, Faradayweg 4-6, 14195 Berlin, Germany

## Abstract

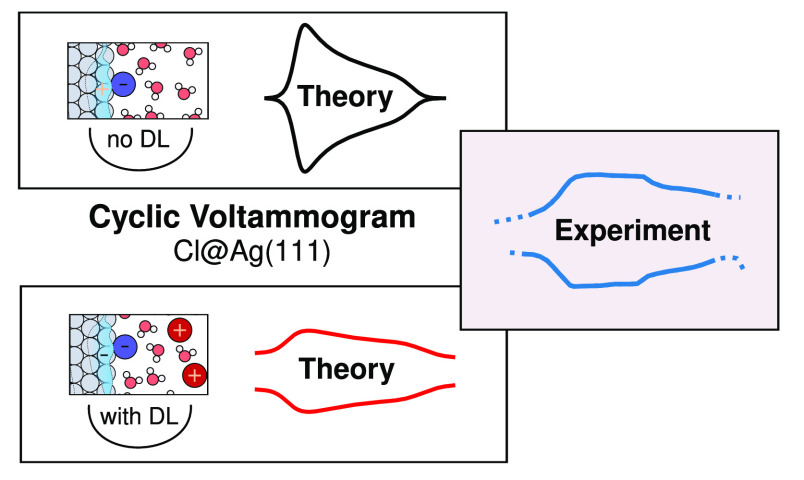

Cyclic voltammograms (CVs) are a central experimental tool for assessing the structure and activity of electrochemical interfaces. Based on a mean-field ansatz for the interface energetics under applied potential conditions, we here derive an *ab initio* thermodynamics approach to efficiently simulate thermodynamic CVs. All unknown parameters are determined from density functional theory (DFT) calculations coupled to an implicit solvent model. For the showcased CVs of Ag(111) electrodes in halide-anion-containing solutions, these simulations demonstrate the relevance of double-layer contributions to explain experimentally observed differences in peak shapes over the halide series. Only the appropriate account of interfacial charging allows us to capture the differences in equilibrium coverage and total electronic surface charge that cause the varying peak shapes. As a case in point, this analysis demonstrates that prominent features in CVs do not only derive from changes in adsorbate structure or coverage but can also be related to variations of the electrosorption valency. Such double-layer effects are proportional to adsorbate-induced changes in the work function and/or interfacial capacitance. They are thus especially pronounced for electronegative halides and other adsorbates that affect these interface properties. In addition, the analysis allows us to draw conclusions on how the possible inaccuracy of implicit solvation models can indirectly affect the accuracy of other predicted quantities such as CVs.

## Introduction

The detailed determination of the reaction mechanism under operation conditions is a major building block to understand and rationally improve electrocatalysts. One prerequisite to this end is the knowledge of the surface structure and composition under applied potential. Cyclic voltammetry is one of the most widespread electrochemical characterization techniques employed for this task. In practice, cyclic voltammograms (CVs) are obtained by varying the electrode potential at a fixed scan rate and measuring the current response of the electrode immersed in the electrolyte solution. The method is thus sensitive to changes in the number of electrons residing at the electrochemical interface, which allows us to infer interface reactions, e.g., electrosorption processes, and concomitant changes in surface composition as a function of the applied electrode potential. In spite of the relevance and indirect nature of this technique, only a limited number of theoretical studies exist that try to quantitatively predict CV curves from first-principles calculations and therewith aid the interpretation of the experimental data^1−10^ (see, e.g., also the excellent review of Li et al.^11^ and the referenced works therein).

This scarcity is even more surprising when recalling that in the limit of small scan rates and thus minimized kinetic effects, CV curves become proportional to the second derivative of the interface free energy with respect to the applied potential. CV simulation and comparison to top-quality experimental data provide thus an intriguing opportunity to assess the quality of the underlying first-principles energetics of the electrified interface. The latter forms the core of an exploding number of *in silico* screening studies to identify improved electrocatalyst materials.^12−17^

**Figure 1:**
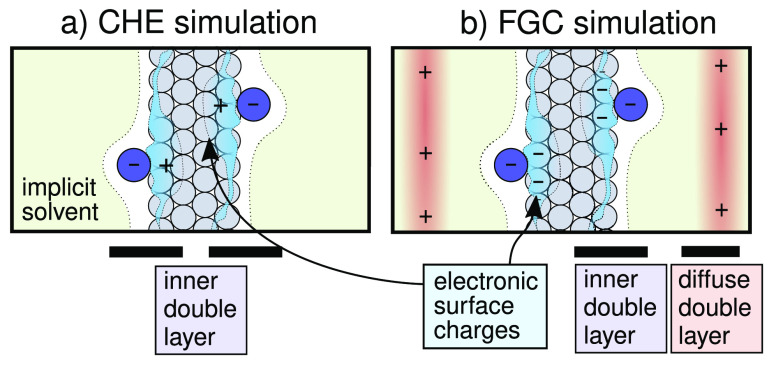
Typical computational setup in implicit solvent models for computational hydrogen electrode (CHE) and fully grand canonical (FGC) simulations. The FGC setup is characterized by the explicit variation of the electronic surface charges as a response to the applied potential (and compensating electrolyte counter charges in the implicit solvent), in contrast to the CHE approximation that can *a priori* only be applied to charge-neutral systems. In the sketched setup without explicit electrolyte solution, the CHE approach thus only allows us to treat charge-neutral inner double layers.

Validating the employed energetics is thus highly pertinent and timely, in particular, as the high demands on computational efficiency in such studies often dictate the use of approximate treatments of solvation and charging effects. A cornerstone in this respect is the computational hydrogen electrode (CHE) approach of Nørskov and Rossmeisl,^18−21^ where the electrochemical potential of the proton–electron pair is related to the corresponding chemical potential of gaseous hydrogen. As a result, the corresponding energetic evaluation can only be applied to systems with an equal amount of protons and (excess) electrons, or in more general terms, to overall charge-neutral interfaces. As typical calculations focus the atomistically resolved first-principles density functional theory (DFT) calculations to the electrode surface, the specifically adsorbed atoms and molecules and at maximum a few solvent molecules, this charge neutrality condition extends only over the inner double layer (DL) (see Figure 1).

In reality, some degree of charge compensation will, however, be provided by solvent screening and the electrolyte ions, leading ultimately to the build-up of the so-called diffuse DL layer (cf. Figure 1). At typical extensions over several nanometers, a full first-principles consideration of this diffuse DL in purely atomistic models is still largely prohibitive, in particular as this generally also implies to appropriately consider the inherent dynamics.^9,22−26^

In such a setup, the generalized CHE (GCHE) approach^27^ can be used to evaluate the respective interface energetics at applied potential conditions, via the use of molecular dynamics simulations and monitoring of the observed work functions. In contrast to the CHE approach, which does not pay attention to the work function, the GCHE correctly uses only CHE-like energy differences for systems, where the system-inherent work function is identical to the applied electrode potential. Ab initio sampling and the introduction of ions in a thin explicit solvent shell, thus indeed allows us to capture DL charging effects, whose accuracy is, however, still limited by the sampling and the achievable ion concentrations and distributions in such all-explicit simulations.

Alternatively, recent advances in coupling periodic DFT codes to implicit solvation models^28−36^ allow nowadays to capture solvent screening and DL effects in a straightforward, albeit continuum way at a low computational cost. An increasing number of theoretical studies following this approach have highlighted the importance of these effects,^29−33,35,37−54^ e.g., for understanding the potential dependence of chemical reaction steps,^32,35,50,55−58^ potential-induced surface reconstructions, or the prediction of surface Pourbaix diagrams.^49,53,59,60^ In such calculations, the total charge of the inner DL is no longer restricted to zero, but can vary with the applied electrode potential. In addition, it was shown that already a second-order approximation to this fully grand canonical (FGC) energetics with a potential-independent interfacial capacitance is very accurate.^43,49,56,61−67^ We refer to this type of approximation to the interface energetics as the CHE + DL approach, as the energetics corresponds identically to the CHE result plus a generic DL energy contribution due to capacitive charging.^61,66,67^

Here, we transfer these recent developments to the context of cyclic voltammetry and present a concise, mean-field *ab initio* thermodynamics-based approach^68,69^ to derive thermodynamic CVs at the CHE and CHE + DL levels of theory. While general CVs, which measure the current–voltage characteristics, are to be simulated with kinetic models,^10^ it has been shown that a thermodynamic treatment can provide accurate predictions whenever the kinetics is of lower importance.^1,9^ This is the case for CVs that are measured within the stability window of the solvent, at very slow scan rates^70^ and for systems, where no faradic side reactions occur. Apart from providing a computationally most efficient first-principles access to CVs, one advantage of this approach is that it allows us to single out the “+DL” effects, i.e., contributions due to the capacitive charging of the DL. This allows us to revisit experimental CVs for Ag(111) electrodes in halide-containing solutions.^71^ The varying peak shapes observed for the different halide ions—Cl^–^, Br^–^, and I^–^—are found to be at variance with CHE model predictions. In contrast, we can fully rationalize them by varying DL contributions due to the different electrosorption valencies (electronegativities) of the adsorbates. Our results thus highlight the decisive role of DL-related energy contributions for understanding experimental CV curves, and vice versa the danger of interpreting CV curves merely in terms of structural and compositional changes in the inner DL.

## Theory

### *Ab Initio* Thermodynamics

In this work, we focus on an ideal-crystalline monometallic electrode composed of species s and offering one type of adsorption site for a single species of adsorbates a that are present as ions in solution. The extension to composite electrodes, several adsorption site types, and multiple adsorbate species is straightforward, but the accumulating sums and indices will make the equations less accessible. All solvent degrees of freedom are furthermore only considered implicitly through the free-energy contributions of a continuum solvent model. In this case, any interface configuration α is fully characterized by the detailed geometric arrangement of the adsorbates on the lattice of adsorption sites and the overall chemical composition, i.e., the number of substrate atoms *N*_s_^α^, the number of possibly charged adsorbate species *N*_a_^α^, and the number of electrons *N*_e_^abs,α^ that reside on the metallic electrode in excess of the charge-neutral pristine electrode surface. *N*_e_^abs,α^ thus corresponds to the number of electrons necessary to compensate for the *N*_a_^α^ adsorbed ions of charge *q*_a_ (, with *e* the electronic charge) plus the number of electrons responsible for the charging of the double layer.

The fundamental quantity in an *ab initio* thermodynamics approach to describe this interface configuration α is the Gibbs excess energy^53,59,67,69,72^
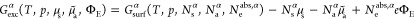
1where *G*_surf_^α^(*T*, *p*, *N*_s_^α^, *N*_a_^α^, *N*_e_^abs,α^) is the extensive Gibbs free energy of the total system containing the interface. *G*_exc_^α^ describes the cost of creating the interface α when taking its constituents from bulk-like reservoirs that are characterized by a chemical potential μ_s_ of the substrate atoms, the electrochemical potential μ̃_a_ of the adsorbate species, and the electrochemical potential of the electrons μ̃_e_ = −*e*Φ_E_ with Φ_E_ corresponding to the electrode potential. Note that the tilde is used to discriminate between the electrochemical potential μ̃ of charged species (in the reservoir) and the chemical potential μ of noncharged species, and in the following, we will drop the explicit dependence on temperature *T* and pressure *p* for ease of notation. The electrode potential Φ_E_ is measured according to electrochemistry conventions, with increasing values away from the zero-reference vacuum level such that, e.g., the experimental standard hydrogen electrode (SHE) lies at +4.44 V on this absolute scale.^73^ The electrochemical potential μ̃_a_ of the ionic species a with chemical symbol A is typically referenced against the experimental equilibrium potential Φ_a,eq_^exp^ of the redox reaction  with known ion concentration *c*_a,eq_ under certain reference conditions, including typically ambient temperature, ions in a 1 M solution, and species A in a standard reference phase.
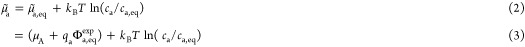
2Here, *c*_a_ is the ion concentration in solution (at nonreference conditions), *k*_B_ is the Boltzmann constant, and μ_A_ is the chemical potential of neutral species A in the reference phase. In the case of halides, as studied here, the reference phases and conditions are the gas phase of diatomic molecules at ambient temperature and 1 bar. The reference chemical potential per particle μ_A_ is thus given by 1/2μ_A_2_(g)_.^21,74^

*G*_exc_^α^ in eq 1 is extensive which is convenient when addressing explicit simulations of interfaces that are performed in periodic supercells with surface area *A* and at integer atom numbers (*N*_s_^α^, *N*_a_^α^). When comparing results obtained in different supercells, it is instead helpful to normalize *G*_exc_^α^ with respect to size. Here, we normalize with respect to the adsorption sites *N*_sites_ and henceforth denote the corresponding intensive Gibbs free energies by lowercase letters. Suitably introducing the excess energy of the clean surface *g*_exc_^clean^ and the average adsorption energy *G̅*_ads_^α^ per adsorbate
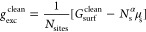
4
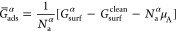
5we thus obtain
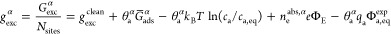
6with θ_a_^α^ = *N*_a_^α^/*N*_sites_ the surface coverage of adsorbates a, measured as the average number of adsorbates per adsorption site, and *n*_e_^abs,α^ accordingly the average number of electrons. Furthermore, all configurations α refer to symmetric slab calculations so that the normalization is trivially defined with respect to the total number of sites offered at both equivalent interfaces.

Minimizing *g*_exc_^α^ with respect to the number of electrons *n*_e_^abs,α^ at fixed composition θ_a_^α^ finally yields the charge-equilibrated excess energy .  defines the cost of creating the interface configuration at a given applied potential Φ_E_^53,67^ and is thus the pertinent quantity for the simulation of thermodynamic CVs. As shown in previous studies,^53,66,67^ can be approximated by analytic minimization of a second-order expansion of *g*_exc_^α^ in *n*_e_^abs,α^. Within this approximation,  decomposes into Gibbs free-energy differences determined at the potential of zero charge (PZC) Φ_0_^α^, plus an additional DL charging contribution *g*_exc_^α,DL^. The PZC Gibbs excess energy term *g*_exc,0_^α,CHE^ can thereby be identified^67^ as the contribution that would be captured in the prevalent CHE approximation.^18−20^ Henceforth, all quantities evaluated at the PZC are denoted with a subscript 0, and all terms that derive from the capacitive DL charging (not captured within the CHE approach) are underlined. As an example, the number of electrons *n*_e_^abs,α^ on the surface results as
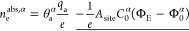
7with *A*_site_ = *A*/*N*_sites_ the surface area per adsorption site and the area-normalized interfacial capacitance *C*_0_^α^ evaluated at the PZC.

Explicitly, the approximation for  then reads
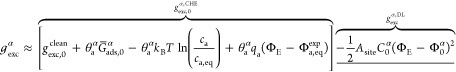
8

### Mean-Field Theory (MFT)

At applied potential Φ_E_ and assuming a sufficiently slow CV scan speed to stay sufficiently close to thermodynamic equilibrium, each configuration α is realized with a probability , where  is the partition function and the sum runs over all possible interface configurations α. These appropriately weighted contributions of different configurations can be explicitly considered through appropriate sampling methods.^4,7,8,65,75−78^ Here, we rely instead on mean-field theory (MFT) as this allows both for a more tractable access when using numerically demanding first-principles calculations for the underlying energetics and for an accessible insight into charge transfer and capacitive contributions to the CVs (see below).

Assuming completely uncorrelated probabilities for the adsorbates to take any of the adsorption sites offered by the crystalline electrode, MFT gives the mean-field charge-equilibrated excess energy in terms of an average adsorbate coverage θ_a_. Within the second-order approximation as before we then have
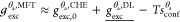
9with the mean-field configurational entropy
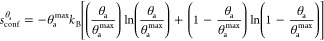
10appropriately normalized with respect to the maximum coverage θ_a_^max^. The CHE and DL terms take the same structure as before
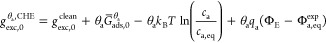
11
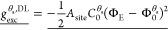
12but now contain the quantities *G̅*_ads,0_^θ_a_^, *C*_0_^θ_a_^, and Φ_0_^θ_a_^ as appropriate averages over all configurations consistent with the average coverage θ_a_. An efficient way to explicitly determine these averages is via the use of special quasi-random structures.^79−81^

The equilibrium coverage θ̅_a_ minimizes  for given Φ_E_ compared to any other hypothetical coverage θ_a_. This minimum condition  results in the implicit sigmoidal equation
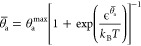
13with , which yields the equilibrium coverage as a function of the potential θ̅_a_(Φ_E_). In the limit of small capacitances *C*_0_^θ_a_^ → 0, all underlined DL terms vanish.

### Thermodynamic CV Simulation

At a sufficiently slow scan rate , we assume the surface charge σ = −*en*_e_^abs^ to be close to its equilibrium value at all times.^1^ The current measured in such a thermodynamic CV is then proportional to the change in this equilibrium charge
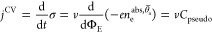
14At a typically constant scan rate, a peak in the CV thus corresponds to a peak in the pseudocapacitance *C*_pseudo_. Within our MFT and second-order ansatz, this pseudocapacitance is approximated as (cf. eq 7)
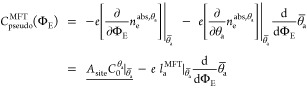
15with the electrosorption valency^67^
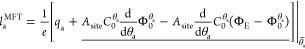
16Note that the explicit potential dependence in the previous equations enters via the potential dependence of the equilibrium coverage θ̅_a_(Φ_E_).

Expression (15) nicely unveils the two expected fundamental contributions to the shape of a CV:^82^ A double-layer charging contribution *A*_site_*C*_0_^θ_a_^ plus a contribution due to adsorption. The prior is generally assumed to vary only smoothly with changing potential and is often called the CV baseline. The latter adsorption contribution results from the actually changing equilibrium coverage (), but equally from changes in the average charge that each adsorbate drags onto the surface as summarized in the classic electrosorption valency *l*_a_^MFT^.^67,82^^67,82^ Only in the limit of vanishing capacitances (vanishing DL terms underlined in the above equations), the electrosorption valency becomes a constant with , e.g., −1 in case of the here considered halide ions, and only in this limit with simultaneously vanishing baseline contribution do we recover the frequently observed interpretation that equates CV curves merely with coverage changes.

We wish to note that these results for thermodynamic CVs are only valid within the stability window of the solvent and without faradic side reactions taking place. Furthermore, the use of the equilibrium surface charges *n*_e_^abs,θ̅_a_^ is only valid when the charging of the double layer as well as the adsorption processes are fast compared to the scan rate.^70^

As an additional note: We chose deliberately the term pseudocapacitance in this work—instead of simply total capacitance—to clarify that our expression is, in particular, suitable for systems where the total interfacial capacitance is given by a double-layer component and an adsorption-related contribution. Typically, double-layer charging can only account for interfacial capacitances of the order of 50 μF/cm^2^ or lower for aqueous solutions. On the other hand, adsorption-related contributions can easily reach several hundreds of μF/cm^2^, whenever dense adlayers of adsorbates are formed. As a result, we think it is helpful to use a distinct term, the pseudocapacitance, in cases where the mere magnitude of the observed total capacitance can only be explained by a combination of charging the DL and specific adsorption processes.

Accurate all-explicit simulation of such processes necessitates very accurate, but computationally still tractable, bond-forming energy models that allow for charge transfer and intelligent sampling methods for solvent and electrolyte as well as adsorbate configurations. This is at present hard to achieve via straightforward (*ab initio*) molecular dynamics with explicit solvent. In fact, to date, most of these challenges are still unresolved. It is thus no surprise that most published studies in this respect^25,83−85^ only address the response of a more or less inert solution with capacitance values of ca. 5–20 μF/cm^2^ and thus do not and cannot address specific adsorption processes which we are interested in here.

## Method and Computational Details

Within the established approach, the simulation of a CV according to eqs 15 and 16 requires (apart from system-specific constants) the quantities *C*_0_^θ_a_^ and Φ_0_^θ_a_^ generally as a function of coverage θ_a_, as well as the equilibrium coverage as a function of the applied potential θ̅_a_ = θ̅_a_(Φ_E_). The latter requires knowledge of *G̅*_ads,0_^θ_a_^.

**Figure 2:**
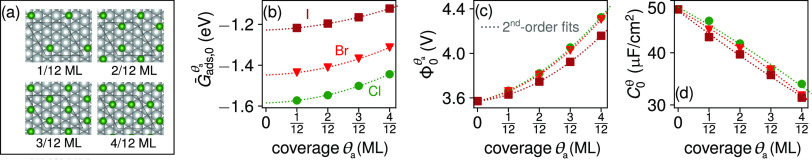
(a) Studied adsorption configurations on the face-centered cubic (fcc) sites of 12^1/2^ × 12^1/2^ surface supercells. (b–d) DFT-determined average adsorption energies per adsorbate *G̅*_ads,0_^θ_a_^, the PZC Φ_0_^θ_a_^, and the interfacial capacitances *C*_0_^θ_a_^ for Cl (green circles), Br (red triangles), and I (brown squares) at different coverages, respectively, including second-order polynomial fits (dotted lines) that are used to evaluate the MFT expressions.

The general workflow to obtain these quantities at predictive quality starts with first-principles electronic structure calculations for specific interface configurations α. They provide all energetics, vibrational and electronic structure information (see below) to compute *g*_exc,0_^clean^, *G̅*_ads,0_^α^, *C*_0_^α^, and Φ_0_^α^ for each configuration, where, in the present application to adsorption at a fixed surface, the term *g*_exc,0_^clean^ is identical to all configurations and consequently drops out in the subsequent coverage-dependent CV simulation. In general, appropriate mean-field sampling of different configurations with identical θ_a_ as described in the Mean-Field Theory section allows us to convert this data into discrete data for *G̅*_ads,0_^θ_a_^, *C*_0_^θ_a_^, and Φ_0_^θ_a_^ at various coverages θ_a_. Here, instead of MF sampling, we choose to approximate the MF result for the low-coverage regime by using single atomic configurations with maximum lateral distance between the adsorbates at a given coverage in the employed supercell (see Figure 2a), as also done in previous studies.^74,86^

Suitable interpolation then yields the three quantities as continuous functions of θ_a_, as illustrated in Figure 2. Substituting this into eqs 9–12 in turn yields the excess energy  equally as an interpolated function of the coverage and as an analytically continuous function of the applied potential. As the equilibrium coverage θ̅_a_ minimizes  at any given potential, analysis of this two-dimensional (2D) free-energy landscape finally yields the relation θ̅_a_ = θ̅_a_(Φ_E_).

For the first-principles calculations, we employ DFT with the PBE exchange–correlation functional^87^ and pseudopotentials from the SSSP library^88^ (v0.7, PBE, efficiency) as implemented in the Quantum ESPRESSO package.^89^ As an implicit solvation model, we use the SCCS implementation of ENVIRON^28,29,90^ with optimized interfacial parameters (ρ_min_ = 0.0013, ρ_max_ = 0.01025, α = β = γ = 0) and a Helmholtz-layer representation of the electrolyte via gaussian-shaped planar counter charges (width: 1 bohr) at a distance of 6 Å from the surface. We have chosen this solvent parametrization and electrolyte representation as it yields good agreement in the interfacial capacitances with the experimental system under study and other systems (see refs (51, 53) and below). A more detailed discussion of the chosen implicit solvent model and its implications are given in the Supporting Information (SI) and the Conclusions section.

Halide CVs on Ag(111) are characterized by two peaks, a broader peak at lower potentials and a very sharp peak at higher potentials (see Figure S2 in the SI). For I and Br these peaks are clearly separated and experiments suggest that the broader peak is due to the electrosorption of up to 1/3 ML, which forms a well-ordered (√3 × √3)R30° structure as observed by *in situ* STM.^71,91^ The latter structure is also observed partly for Cl^71,92^ suggesting the importance of a well-ordered 1/3 ML coverage for all three halides. The sharp peak at higher potentials (see Figure S2 in the SI) is related to adsorbate structures with higher coverages (e.g., 3/7 for Br or 0.5 for Cl).^71^ In this work, we are interested only in simulating the broad peak at lower potentials and thus choose a maximum coverage of 1/3 ML (θ_a_^max^ = 1/3) for all halides. Furthermore, we use equivalent adsorbate structures for all halides, namely, adsorption at the fcc hollow sites of Ag(111), modeled in  supercells with an area per site *A*_site_ = 7.398 Å^2^ at the optimized PBE lattice constant. These cells allow us to compute five different adsorbate coverages from 0 to 4/12 ML (cf. Figure 2a). We consciously chose to treat all halides on the same footing, as it removes possible artifacts when varying structures, compositions and the interpolation scheme and enables thus a consistent comparison of the impact of the varying DL energetics in the description of halide adsorbates. In the SI, we also included a discussion of the observed surface charges from the experiments and from our simulations. With the total integrated charge below the CV peaks as a proxy for the maximum surface coverage, these results support a maximum coverage of 1/3 ML as a reasonable choice (cf. Figure S8).

All calculations are in a symmetric slab setup, consisting of six Ag layers and with a separation to periodic images of at least 17 Å. In all structures, the position of all adsorbates and Ag atoms apart from the central two layers are fully relaxed until residual forces drop below 0.1 eV/Å and total energy variations between consecutive steps below 0.5 meV/adsorbate. Differences in results with a stricter force threshold of 0.02 eV/Å are < 3 meV/adsorbate. Density and wave function cutoffs are 360 and 45 Ry, respectively, and Brillouin zone integrations are performed using Γ-centered (4 × 4 × 1) Monkhorst–Pack meshes and a cold smearing^93^ of 0.02 Ry.

Following the standard *ab initio* thermodynamics approximation,^69,72^ we compute the average adsorption energy per adsorbate at the PZC (cf. eq 5) as
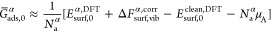
17where *E*_surf,0_^α,DFT^ and *E*_surf,0_^clean,DFT^ are the noncharged, 0 K DFT total energies of the adsorbate-covered and clean slabs, respectively, and Δ*F*_surf,vib_^α,corr^ corrects for the Helmholtz free-energy contributions of the surface vibrational modes of the adsorbed species (see the SI). The reference chemical potentials μ_A_ for the halogens at standard conditions (298 K, 1 bar) are determined from the chemical potential of gas-phase molecules (μ_A_ = 1/2μ_A_2_(g)_) as given by the DFT energy of the relaxed, isolated biatomic molecule with added ideal-gas-like free-energy contributions. The corresponding reference potentials Φ_a,eq_^exp^ are taken from literature standard reduction potentials as summarized in Table 1, valid for 1 molar solutions at 298 K. The DL-related quantities, *C*_0_^θ_a_^ and Φ_0_^θ_a_^ at the PZC, are obtained within the harmonic approximation to the fully grand canonical (FGC) ansatz^46,48,53,57,66,67^ by finite surface charging (eight nonzero, net surface charges) as detailed in the SI. Test calculations at increased computational settings indicate a numerical convergence of the thus obtained average adsorption energies, PZCs, and interfacial capacitances of ±0.005 eV, ±0.02 V, and ±0.5 μF/cm^2^, respectively. A test on adsorption energies in vacuum at low coverages yields differences from reported literature values less than 25 meV.^74,92^

**Table 1:** Literature^86,94,95^ Reference Potentials Φ_a,eq_^exp^ for Halogens, and Electrode Potentials of the Standard Hydrogen Electrode (SHE) and Saturated Calomel Electrode (SCE) on an Absolute Scale

reference and electrode potentials in V				
Φ_Cl,eq_^exp^	Φ_Br,eq_^exp^	Φ_I,eq_^exp^	Φ_SHE_^exp^	Φ_SCE_^exp^
5.80	5.53	4.98	4.44	4.68

Figure 2b–d compiles the computed average adsorption energy per adsorbate *G̅*_ads,0_^θ_a_^, the PZC Φ_0_^θ_a_^, and the interfacial capacitances *C*_0_^θ_a_^ for the three considered adsorbates on Ag(111) together with a second-order polynomial interpolation, which allows us to derive the continuous coverage dependencies. The corresponding numerical values can be found in the SI. For bare Ag(111), previous work has shown that the present computational setup yields interfacial capacitances in good agreement with experiment.^51^ However, some error in the PZC exists, 3.57 vs 3.99 V (exp).^53^ We therefore add a constant shift of 0.42 V to all first-principles-derived PZCs, to match the experimental value for clean Ag(111). Such a correction is typically necessary, as current purely implicit solvent models are not able to accurately describe PZCs across different substrate materials.^53,96^

## Results and Discussion

### Cyclic Voltammograms

**Figure 3:**
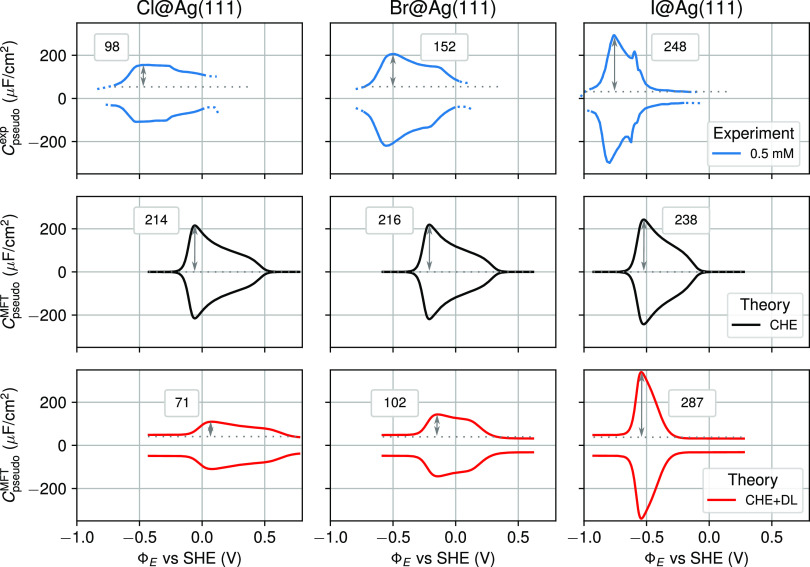
(Top) Experimental cyclic voltammograms (CVs) by Foresti et al.^71^ in the potential range of halide electrosorption (*c*_a_ = 0.5 mM) on Ag(111) (the potential was shifted from the experimental SCE scale to the SHE scale using the values of Table 1). (Middle) Corresponding simulated CVs for the CHE approach. (Bottom) Simulated CVs for the CHE + DL approach. All plots show the scan-rate-independent pseudocapacitance (cf. eq 14 and text for details on the performed normalization for the experimental pseudocapacitances), and include numerical values for the electrosorption-related peak heights (gray arrows; baseline currents indicated by dotted, horizontal lines).

The detailed and consistent set of experimental data provided by Foresti et al.^71^ for halide electrosorption on Ag(111) provides an ideal benchmark for the established framework of computing thermodynamic CVs and related quantities like the electrosorption valency. Figure 3 (top) reproduces these experimental CVs for Cl^–^-, Br^–^-, and I^–^-containing solutions (*c*_a_ = 0.5 mM) on the SHE scale, digitized in the relevant halide-electrosorption region from ref (71) (full CVs in the SI). The experimental currents *j* were normalized to *j*^ref^, the current before the obvious onset of electrosorption at −0.75 V (Cl, Br) and −0.95 V (I) vs SHE. As *j*^ref^ at these potentials is solely related to capacitive DL charging, such a normalization allows us to assign the corresponding pseudocapacitance value  (cf. eq 14), where *C*^ref^ is the DL capacitance of the pristine surface in the respective solution. The latter can be determined from the experimental charge vs potential relation for the clean electrodes (see the SI for more details). The obtained values are 52 (Cl), 49 (Br), and 39 (I) μF/cm^2^, all very close to the value derived for our implicit solvent model (48.3 μF/cm^2^). The slight variations across the experimental systems stem from the natural variation in solution properties when exchanging the halide-ion type,^57^ which is not accounted for in our theoretical approach. The nearly perfect symmetry of the CVs indicates that a thermodynamic treatment, as done here, is indeed applicable.

In Figure 3 (middle and bottom), we report the corresponding theoretical CVs from the CHE and CHE + DL approaches based on eqs 15 and 16. A first general discrepancy between experiment and theory is the overall alignment of the CVs on the potential axis. The rather constant shift of all theoretical CVs to higher potentials indicate too weak adsorption energies. The origin might be manifold: While we have simulated electrosorption as a simple adsorption process, a more realistic description would rather treat it as a substitution reaction of adsorbed water with an adsorbed halide anion. Own test calculations of a corresponding process on other systems with explicit static water, similar to that in ref (97), showed, however, that such a method can introduce significant errors due to the extreme sensitivity of the results to the used water structure^98^ and it is thus hard to assess if the simplified description of the electrosorption process stands behind the error in the absolute peak position. In general, approximate DFT exchange–correlation functionals such as PBE underestimate the formation energy for bulk halides and oxides by ca. 300–400 meV per halogen/oxygen.^99−101^ This error in the bond-formation energy likely translates to spurious, roughly constant shifts in absolute adsorption energies, as can readily be validated by repeating the calculations with different DFT functionals. Consistent errors like these can be corrected by shifting the reference energies of the gas-phase references as, e.g., done with high success in many high-throughput databases.^99,102^ Note that such a correction would not affect the overall peak shape though but only its position. An evaluation with the equally popular, semilocal revPBE functional, which shows typically more positive adsorption energies,^103^ and less accurate work functions and structural properties,^104,105^ yields theoretical CVs that agree less with the experiments than the PBE calculations (see Figure S7 in the SI). The inability of semilocal functionals to adequately capture halide binding is as intriguing as it is annoying; however, we will accept in the following this general misalignment and focus on the detailed simulation of the CV peak shapes.

Already the CHE approach (Figure 3, middle) captures the significant peak broadening with the characteristic butterfly shape. It does not account for any ion specificity though and predicts quite similar peak heights, widths, and shapes for all three halide ions. In contrast, the experimental CVs show a clear trend from Cl over Br to I, with a continuing contraction of the peak together with a concomitant increase in the maximum pseudocapacitance (cf. reported peak heights in Figure 3). Gratifyingly, the CHE + DL approach nicely yields the trend and naturally even features the baseline contribution. In particular, this direct comparison of the CHE and CHE + DL results thus reveals quite different physical contributions to the overall experimental CV shape, i.e., from coverage-dependent adsorption energies and double-layer charging. This immediately highlights the danger of the common interpretation of these shapes merely in terms of the prior energetics. Such interpretation would likely have rationalized the wider Cl CV peak with stronger repulsive adsorbate–adsorbate interactions than between the other halides. Instead, the increasingly contracted, and thus higher, Br and I CV peaks derive clearly from energy contributions due to capacitive DL charging, as this contraction is only captured by the CHE + DL approach. In the SI, we also report on the integrated pseudocapacitances, which corresponds to the interfacial charge as a function of the potential which was also measured in the experiments. These results equally show that the different behavior of halides can only be explained within the CHE + DL model.

In the following sections, we will further elaborate and analyze in detail the origin of these observed variations.

### Equilibrium Surface Coverages

**Figure 4:**
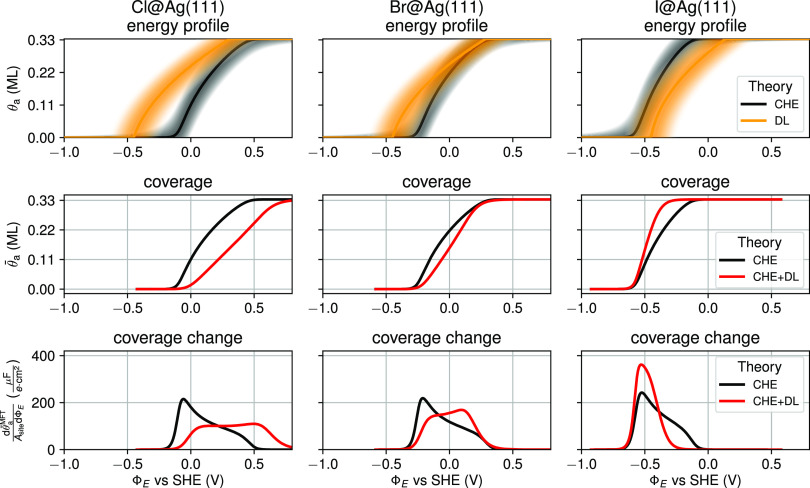
Analysis of the coverage-dependent contribution to the CVs in Figure 3. (Top) 2D energy landscape for the CHE excess energy (*g*_exc,0_^θ_a_,CHE^ – *Ts*_conf_^θ_a_^, black) and the DL term (*g*_exc_^θ_a_,DL^, orange) (cf. eq 9). The solid lines follow the extremal value at fixed Φ_E_, while the diffuse range indicates the energy contour up to +(-)5 meV/site for the convex (concave) CHE (DL) energy profile. (Middle) Equilibrium surface coverage θ̅_a_ as extracted from the 2D energy landscape. At the CHE level (black line); this is simply the extremal ridge shown as a black line in the top panel. At the CHE + DL level (red line), this is the extremal ridge of the 2D landscape resulting after summing the CHE and DL contributions. (Bottom) Area-normalized derivative  determining the coverage-dependent contribution to the CV peak shape (see text).

As already noted, in the prevalent picture, the CV shape results foremost from the variation of the equilibrium surface coverage with applied potential. In eq 15, this is reflected through the proportionality of the adsorption-dependent part of the pseudocapacitance with . An assumed coverage-independent average adsorption energy per adsorbate would lead to a Langmuir adsorption isotherm, which increases quickly from zero to maximum coverage around an applied potential (Φ_E_ – Φ_a,eq_^exp^) that corresponds to the constant value of −*G̅*_ads,0_^θ_a_^/*q*_a_ (for *c*_a_ = *c*_a,eq_) and with a width solely dictated by configurational entropy. In the derivative, this gives rise to a simple, narrow peak in the CV. A linearly varying adsorption energy would instead yield a Frumkin isotherm and in turn a CV peak with altered width and shape, yet still without substructure. In the present case, the mutual electrostatic repulsion of the adsorbed halides gives rise to a quadratically increasing^92^*G̅*_ads,0_^θ_a_^ (cf. Figure 2b). As shown in Figure 4 (middle and bottom), this leads to an intuitive and almost identical adsorption isotherm for all three halides at the CHE level: the onset of electrosorption is characterized by a steep initial coverage increase, which levels off continuously until the maximum coverage is reached at approximately 0.6 V above the onset potential. In the derivative also shown in Figure 4, this then gives rise to the characteristic shoulder, leading to the butterfly-type CV peak shape.

Interestingly, the DL contribution captured in the CHE + DL approach also affects this coverage-controlled part of the peak shape, i.e., this contribution also affects the potential dependence of the equilibrium surface coverage. As shown in Figure 4, adsorption occurs over a significantly broadened potential range for Cl and a significantly narrowed range for I compared to the corresponding CHE adsorption isotherms, while the potential range is barely affected for the intermediate case of Br. In the derivative, this then already yields the increasing contraction of the peak width from Cl over Br to I that is also seen in the experimental CVs (cf. Figure 3). This different effect of the DL contribution on θ̅_a_(Φ_E_) can directly be traced back to the different position of the extremal ridge of the corresponding *g*_exc_^θ_a_,DL^ free-energy term relative to the extremal ridge of the (*g*_exc,0_^θ_a_,CHE^ – *Ts*_conf_^θ_a_^) CHE term. In the 2D excess energy landscape shown in Figure 4, the maximum ridge of the prior concave DL term lies at lower potentials compared to the minimum ridge of the latter convex CHE term in the case of Cl. For Br, both ridges almost coincide, while for I, the DL ridge lies at higher potentials. This different relative position changes the equilibrium coverage defining a minimum ridge of  that results as the sum of these two energy terms at the CHE + DL level.

In terms of physics, the change of relative ridge position arises a consequence of the varying reactivity of the three halides (which determines the position of the minimum CHE ridge) (cf. Figure 2b), while the PZC (which determines the position of the maximum DL ridge) shows barely any ion specificity (cf. Figure 2c). Both the adsorption energies and the solvation strengths thereby follow the expected electronegativity trend, with Cl showing the strongest reactivity. As the solvation strength and electron affinity (which relate directly to the experimental reference potentials Φ_a,eq_^exp^ in Table 1) increase even more over the halide series than the adsorption energies, the onset potential for electrosorption as determined by the difference of adsorption strength and reference potential actually exhibits an opposite trend, i.e., it shifts from Cl over Br to I to consecutively lower potentials. The largely invariant PZC, on the other hand, reflects similar adsorbate dipole moments for the three halides, with small variations arising from opposing trends in electronegativity (and thus electrosorption valency, see below) and ionic radius.^106,107^

With electrosorption thus taking place at potentials above (Cl) and below (I) the PZC, the adsorbates experience an electrostatic field in the DL of opposite direction. The consequently reversed dipole–field interaction shifts the peaks in opposing directions and acts effectively like reversed lateral interactions between the adsorbed halides, stretching the adsorption isotherm over a larger potential range as compared to the CHE result in the case of Cl, while contracting it in the case of I.

### Electrosorption Valency

The analysis of the DL effects on the adsorption isotherm in the preceding section rationalize the increasing contraction of the CV peak from Cl over Br to I seen in the experimental CVs. However, when comparing the corresponding images in Figure 4 (bottom) with the full CV simulations in Figure 3, it is clear that this is not yet the full story. In particular, for Cl and Br, the peak substructure is not properly reproduced, with the spectral dominance of the lower-potential shoulder only correctly captured by the full CHE + DL simulations in Figure 3. This is because the pseudocapacitance in eq 15 is not only proportional to , but equally to the electrosorption valency *l*_a_^MFT^. This valency only reduces to a mere constant *q*_a_/*e* at the CHE level, i.e., −1 for all here considered halides. In contrast, at the CHE + DL level, the capacitance-dependent terms in eq 16 make this average charge that each adsorbate effectively drags onto the surface also coverage- and ion-dependent.

**Figure 5:**
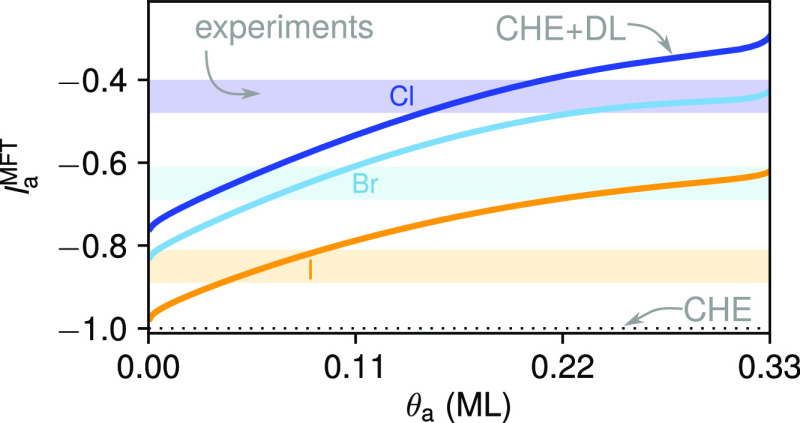
Ion and coverage dependence of the electrosorption valency *l*_a_^MFT^. The potential-dependent, latter term in the CHE+DL approximation (eq 16) is evaluated at the respective equilibrium potential for the given coverage, which leads to minor differences with previously published results^67^ based on the experimental potentials. The CHE (horizontal dotted line) and the experimental results^71^ (colored horizontal bars) are included as well.

Figure 5 shows this ion and coverage dependency of the electrosorption valency as computed at the CHE + DL level. Notably and as discussed in previous work,^67^*l*_a_^MFT^ is consistently larger than −1 for all halides and all coverages, and agrees well with the experimental values.^67,71^ The actual charge transferred upon electrosorption of a halide ion is thus significantly less than the full formal charge assumed *a priori* at the CHE level. The effect becomes even more clear, when comparing simulated and experimental surface charges, as done in the SI, where the experimental variation of the total integrated charge below the CV peak can be captured nicely within the CHE + DL model (see Figure S8).

Again, the trend over the three halides thereby follows electronegativity, with the most electronegative Cl ions releasing the least amount of charge to the electrode. Even though, if this noninteger electrosorption valency was coverage-independent, it would still merely renormalize the pseudocapacitance and leave the CV shape unaffected. Instead, *l*_a_^MFT^ becomes continuously more positive with increasing coverage (cf. Figure 5). At higher coverages, less charge is thus transferred per electrosorbing ion, which thus induces an increased damping of the higher-potential part of the CV peak where the maximum coverage is approached. Effectively, it is precisely this damping that reduces the weight of the higher-potential shoulder of the simulated CVs and finally leads to the good agreement of the full CHE + DL simulated CVs in Figure 3 with the experimental data.

## Conclusions

Recent work has established fully grand canonical DFT calculations with an implicit solvent model as a computationally most efficient way to approximately capture diffuse DL layer effects at electrified interfaces from first principles. In this work, we have extended this approach to the context of cyclic voltammetry, specifically by integrating it into an *ab initio* thermodynamics framework and employing a mean-field approximation for the adsorbate configurations at the electrode surface. Requiring only a limited number of first-principles calculations to determine average adsorption free energies, points of zero charge, and interfacial capacitances, thermodynamic CVs can in this way readily be simulated with (CHE + DL) and without (CHE) consideration of the diffuse DL layer. The direct comparison of the two levels of theory thus allows us to explicitly single out capacitive charging effects on the simulated CV curves.

The established framework is without doubt highly effective, with the most notable inherent approximations being the neglect of kinetic effects, the mean-field averaging and the reliance on semilocal DFT calculations with a continuum solvation model. On the other hand, apart from the computational efficiency, it is noteworthy that starting from an abstract free-energy landscape for electrified interfaces the mathematical derivation leads to equations of appealing simplicity, in which important fundamental quantities like the CV baseline current or electrosorption valency emerge naturally. These results underline the importance and value of mean-field models as they can provide interpretable, analytic relations between observations and basic descriptors of the electrochemical interface, which are not easily accessible from other, more accurate methods, such as sampling of a voltage-dependent cluster expansion Hamiltonian,^65,78^ or fully explicit simulations.

The showcase application to CVs from Ag(111) electrodes in halide-anion-containing solutions demonstrated that semiquantitative agreement with existing experimental data can only be achieved when explicitly considering DL effects. This is particularly true for the peak shapes and especially the trend of the varying peak shapes over the halide series, which is intriguingly well reproduced at the CHE + DL level.

Note, that the similarity of the adsorption energetics at the PZC, which is reflected in the similarity of the CHE-derived CVs, is in perfect agreement with chemical intuition as we used consistent structures and expect similar interactions for all halides, dominated by similar electrostatic adsorbate–adsorbate and bonding interactions with the substrate, also for more refined interface models with, e.g., some explicit water. As a result, the variations between the experimental CVs are certainly surprising within the prevalent interpretation which relates CV curves solely to changes in adsorption geometry or surface coverage. On the other hand, the straightforward explanation of these differences by an interface model that includes DL charging, confirms that these are indeed relevant driving forces in the studied systems and that the magnitude of these effects is already described correctly at the employed approximate level of theory. As evident from the provided formulas, the corresponding DL effects on the CV curves will be particularly pronounced for adsorbates that induce significant changes in the work function and/or the interfacial capacitance, such as in the case of the electronegative halides, considered here.

The major remaining discrepancies of the theoretical predictions are in the form of a constant offset on the potential scale and a slight overestimation of DL effects, as a close inspection of the CV peak heights in Figure 3 and also of the total surface charges in Figure S8 in the SI reveals. As discussed already before, the peak position is related to insecurities in the determination of absolute adsorption energies, which remains a general, unsolved problem for the *ab initio* community, as it can be related to the studied interface model, the treatment of solvation, and the DFT functional. On the other hand, the magnitude of double-layer effects is mainly related to the work function change (and thus adsorbate dipole) and the interfacial DL capacitance. As the DL capacitance of our implicit model is in rather good agreement with the experimental values, we think that the overestimation of such effects in our calculations hints at an overestimation of the work function change due to adsorption. Such an error might be related to our choice using a fully implicit model. Mobile, explicit interfacial water might likely provide better shielding and dipolar response than the simple implicit model, thus leading to a dampening of the observed work function changes. Interface models that include (partly) explicit water are also expected to improve upon other interfacial properties, e.g., PZCs,^35,56,96^ which implicit solvent models evidently struggle to reproduce.^51,53,96,108^ In addition, such implicit/explicit hybrid models might likely be more accurate in describing the coverage dependence of the interfacial capacitance. The observed reduction with increased coverage (cf. Figure 2d) is a generic behavior of fully implicit models, also observed for other adsorbates, as it derives from the mere distance increase between the dielectric and the metallic surface in adsorbate-covered regions^109^ (see Figure S3 and the discussion in the SI). A comparison to the experimental charge vs potential curves indicates rather unchanged interfacial capacitances (cf. derivatives before and after adsorption in Figure S8 in the SI), hinting thus at another inherent accuracy limitation of implicit models. In addition, different models for the electrolyte might induce some variations of the interfacial capacitance with the potential,^51,53,108,110^ which can not be captured by construction in our model. Numerical experiments confirm though that these are not important for the studied halides.

While all of these mentioned uncertainties seem highly problematic at first sight, it is one major strength of the present mean-field model that it allows us to assess their effect on relevant quantities such as, e.g., CVs. This insight will be highly relevant for the future improvement and error estimation of solvation models and enable the reflected choice of appropriate interface models and modeling schemes to achieve certain target resolutions and accuracies, based on prior quantitative analysis of simplified models as the one presented here.

## Supporting Information Available

Full experimental CV curves, a discussion of the used implicit solvent model, simulated CVs based on revPBE calculations, analysis of theoretical and experimental surface charges, numerical values for adsorption energies, vibrational free-energy corrections, work functions, and interfacial capacitances (PDF)
