# Supplementary material for: Thermodynamic Cyclic Voltammograms Based on Ab Initio Calculations: Ag(111) in Halide-Containing Solutions
Source: J Chem Theory Comput. 2021 Feb 19;17(3):1782–94. doi: 10.1021/acs.jctc.0c01166 (PMC8023662; doi:10.1021/acs.jctc.0c01166)
Supplement: Supplementary file 1 — ct0c01166_si_001.pdf [file ct0c01166_si_001.pdf]

Supporting Information: Thermodynamic cyclic voltammograms based on *ab initio* calculations: Ag(111) in halide-containing solutions

Nicolas G. Hörmann*,†,‡ and Karsten Reuter‡

†*Chair of Theoretical Chemistry and Catalysis Research Center, Technische Universität München, Lichtenbergstr. 4, 85748 Garching, Germany*

‡*Fritz-Haber-Institut der Max-Planck-Gesellschaft, Faradayweg 4-6, 14195 Berlin, Germany*

E-mail: nicolas.hoermann@tum.de

Digitized Experimental Results and Normalization Procedure

Digitized experimental results by Foresti et al.[S1] are plotted in Supplementary Fig. S1 and Fig. S2. Supplementary Fig. S1 shows the experimental surface charge vs potential curves for pristine Ag(111) surfaces in the different halide ion solutions. The derived average interfacial capacitances $\bar{C}$ were taken as references C^{ref}, to map the experimental CVs (Supplementary Fig. S2) onto scan-rate-independent pseudo capacitances.

The experimental CVs by Foresti et al.[S1] (Supplementary Fig. S2) show sharp peaks at the higher potential end which is due to formation of silver halide surface layers. These regions are not plotted in the main text for reasons of clarity. The CV-derived pseudocapac-

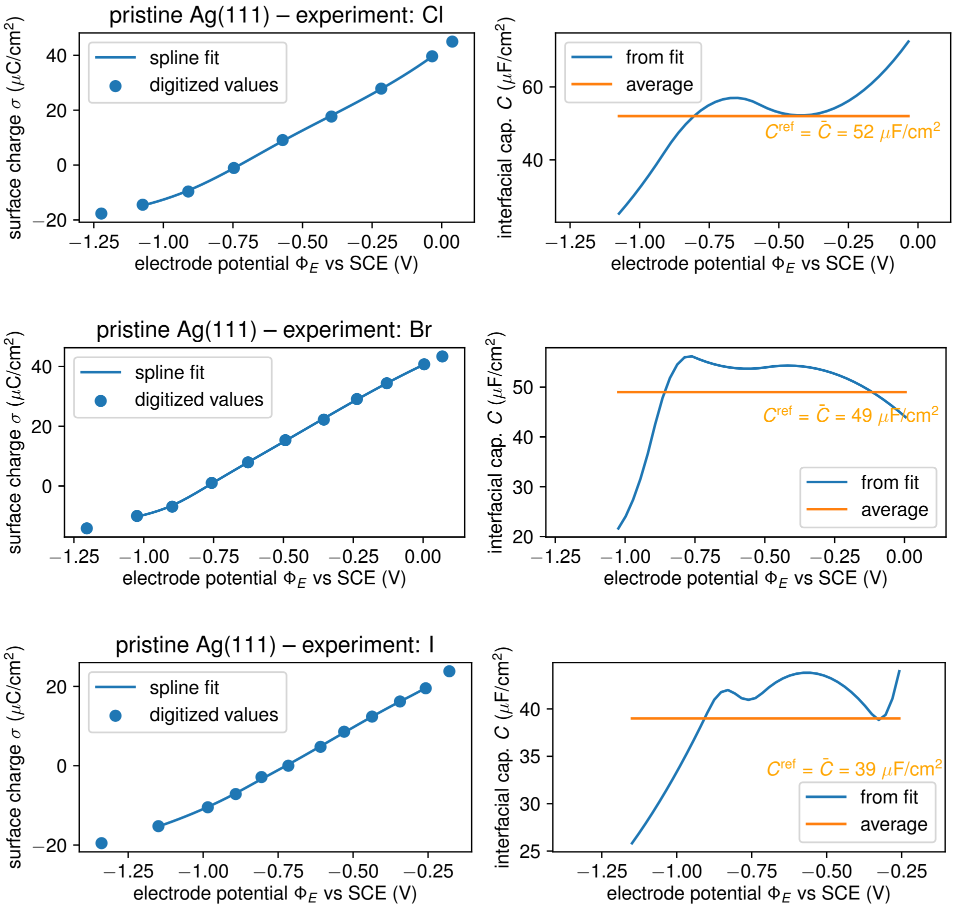

Figure S1: Digitized experimental surface charge σ vs potential Φ_E of pristine Ag(111) surfaces in different halide ion solutions. Data from experiments by Foresti et al[S1]. Average interfacial capacitances $\bar{C}$ were obtained by the averaged, numerical derivative of interpolated spline functions (with $C = \frac{\mathrm{d}}{\mathrm{d}\Phi_E}\sigma$). Averages were taken over regions with nearly linear charge vs potential curves (c.f. plotted blue lines), yielding values of 52, 49, and 39 $\mu\mathrm{F/cm}^2$ for the Cl, Br and I experiments, respectively. The so-determined average values $\bar{C}$ were taken as references C^{ref}, to map the experimental CVs on the scan-rate-independent pseudo capacitance curves $C^{\mathrm{exp}}_{\mathrm{pseudo}}$ reported in the main text (see also Supplementary Fig. S2)

itance values $C^{\mathrm{exp}}_{\mathrm{pseudo}} = \frac{j}{j^{\mathrm{ref}}} C^{\mathrm{ref}}$ as reported in the main text were obtained by normalizing the experimental currents j to reference currents j^{ref} – the current before the obvious onset of electrosorption, marked with orange stars in Supplementary Fig. S2 – and subsequent multiplication with C^{ref}. As j^{ref} at these points is solely related to capacitive DL charging, we use the averaged double layer capacitances $C^{\mathrm{ref}} = \bar{C}$ for the clean surfaces as determined in Supplementary Fig. S1.

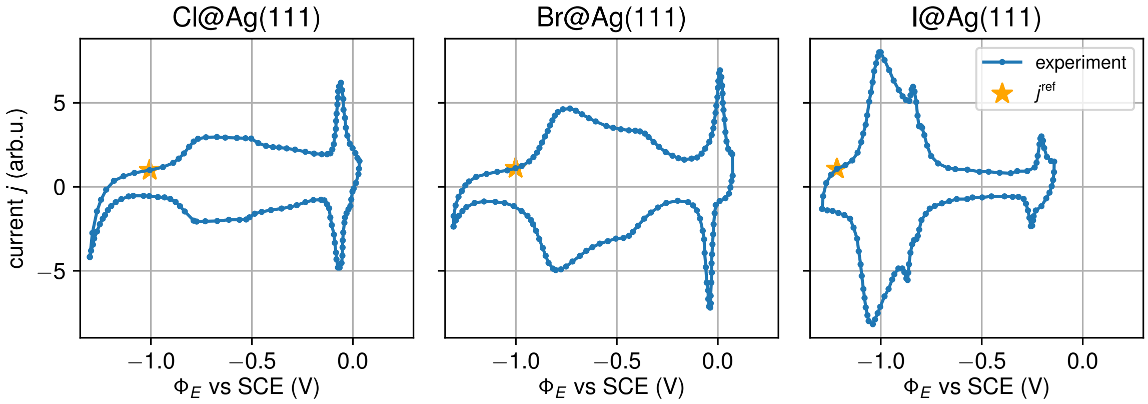

Figure S2: Digitized full CVs from Foresti et al.[S1]. The sharp peaks at the higher potential end are due to formation of silver halide surface layers and not plotted in the main text for reasons of clarity. The orange star marks the reference current j^{ref} before the onset of halide electrosorption, which were used in combination with the capacitance values C^{ref} of the pristine slabs (see Supplementary Fig. S1) to transform experimental CV currents j to scan-rate independent pseudocapacitance curves $C^{\mathrm{exp}}_{\mathrm{pseudo}}$ reported in the main text.

Additional computational details and results

Implicit solvent model

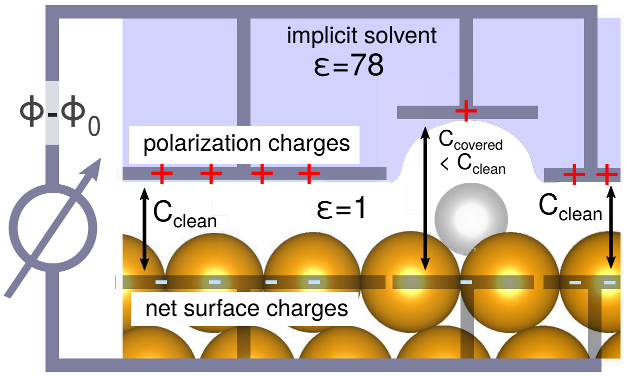

Figure S3: Sketch of the interfacial capacitance in implicit models for high electrolyte concentrations. Under these conditions the potential drop within in the solvent can be neglected as it is small compared to the one that occurs across the vacuum gap between quantum-mechanical region and the onset of the dielectric in the implicit model with $\epsilon \approx 78$. Thus the interfacial capacitance is dominated by the vacuum-solvent gap and thus the parametrization of the dielectric onset in the implicit model. The sketch allows as well an intuitive understanding of the reduction in the (average) interfacial capacitance for adsorbate-covered surfaces in implicit models, as observed.

As stated in the main text, we chose as an implicit solvation model the SCCS implementation of ENVIRON[S2–S5] with optimized interfacial parameters ($\rho_{\mathrm{min}} = 0.0013$, $\rho_{\mathrm{max}} = 0.01025$, $\alpha = \beta = \gamma = 0$) and a Helmholtz-layer representation of the electrolyte via gaussian-shaped planar counter charges (width: 1 bohr) at a distance of 6 Å from the surface. This solvent parametrizations and electrolyte representation yields good agreement in the interfacial capacitances with the experimental system under study and other systems ($\approx 40-50$ μF/cm^2 for clean low index Ag and Pt surfaces, see Refs. S5,S6).

Note that all implicit solvent models behave approximately like two capacitors in series[S7,S8]: the first capacitor is characterized by the vacuum gap between the quantum-mechanical region and the onset of the dielectric, which is where polarization charges screen effectively 99% (1-1/ϵ, with ϵ the dielectric constant of water as used in the implicit model, $\epsilon \approx 78$) of the interfacial field. The second capacitor is between the dielectric onset and

the position of the electrolyte counter charges, which are, for reasonable models, confided to the dielectric region. As a result, it is the potential drop between quantum-mechanical region and the onset of the dielectric that puts an upper limit to the total DL capacitance in implicit models, independent on the electrolyte model (cf. also Fig. S3).

Standard parametrizations of SCCS implicit solvent model (e.g. to reproduce the solvation energy of water, or uncharged and positive small molecules) yield interfacial capacitances between 10-20 μF and thus below the values of the experiments studied in this work, independent on electrolyte representation.

Thus it is only possible to approximately reproduce experiments with larger interfacial capacitances by reparametrizing charge-density thresholds for the dielectric onset in the SCCS model. Note that our parameters are in fact approximately the average between parameters used to optimally describe the solvation of anionic, cationic and neutral solvent molecules, thus likey describing all of these good (in average) (cf. Ref. S6).

Turning now to the electrolyte model: A simple Guy-Chapman picture (= solution to the 1D linearized Poisson-Boltzmann model) yields an interfacial capacitance equivalent to a plate capacitor model with a fixed distance between surface and electrolyte screening charges. This fixed distance is related to the electrolyte concentration and extremely small ($\mathscr{O}$(Å)) for high electrolyte concentrations, which we try to mimic by our chosen planar counter charges at a distance of 6 Å from the surface. In this case the interfacial capacitance does not exhibit a strong dependence on the applied electrode potential (see e.g. Ref. S9 and Fig. S5 here).

For high electrolyte concentrations, also all other reasonable electrolyte models (e.g. modified Poisson-Boltzmann, with or without Stern layer[S8,S10]) that confine the electrolyte charge to the implicit region (dielectric constant $\epsilon \approx 78$), will lead to electrolyte counter charges that are located in close proximity to the interface region ($\mathscr{O}$(Å)). Due to the fact that all fields in this region of space are scaled down by a factor of $\approx 1/78$ the total potential drop in this region of space is scaled down by an according factor.

As a result, the total potential drop across the DL in an implicit model at high electrolyte

representations consisting first of the potential drop in the discussed vacuum gap and the consecutive 'solvent' gap up to the electrolyte counter charges, is fully dominated by the former[S7]. As a result of the small value of the latter the details of the electrolyte model are unimportant for the observed total capacitance. This picture of the interfacial capacitance also explains the observed reduction when the surface becomes adsorbate-covered and thus there is an effective increase in the vacuum gap (cf. Fig. S3).

It is worth noting, that this picture changes when studying systems at low electrolyte concentrations, where the potential drop within the dielectric region becomes sizable, and thus making the specifics of the electrolyte model visible and significant. For more details, we refer the reader to the discussions in Refs. S5,S11. In general, more complex electrolyte models e.g. based on a modified Poisson-Boltzmann model and with or without Stern layer, lead to a potential dependence in the interfacial capacitance, which however is mostly pronounced for small electrolyte concentrations[S5,S11], and (thus) small capacitances.

Here, we use only a quadratic approximation to the potential dependence for the interfacial energetics, and thus a priori neglect all higher order variations, which is why we also rather want to avoid these in the implicit model calculations.

In summary, our specific choice of implicit model parametrization and electrolyte representation is thus based on the fact that we indeed try to model a situation at high electrolyte concentrations (note the halide concentrations in Ref. S1 are only 0.5 mM, however all experiments are done with 0.15 M NaOH, 0.1 M KPF_6 background electrolyte), the fact that there is no (clear) evidence for a very complex interfacial double layer capacitance (see Figure S1) and the fact that we aim at only discussing the effects of an assumed potential-independent interfacial capacitance.

Vibrational free energy correction

The free energy corrections $\Delta F^{\alpha,\mathrm{corr}}_{\mathrm{surf,vib}}$ due to the vibrations of the adsorbed halides are determined from the concentration-dependent, DFT-derived vibrational spectrum of the

adsorbates only and are plotted in Supplementary Fig. S4.

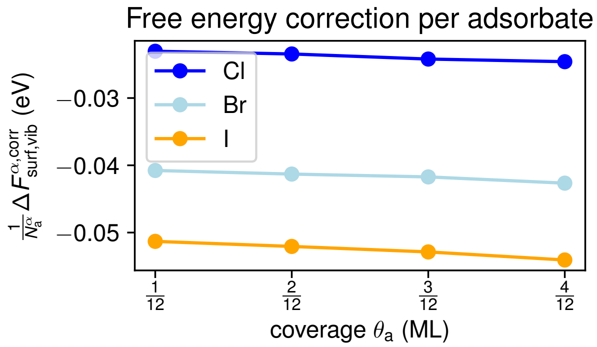

Figure S4: Numerical values of $\Delta F^{\alpha,\mathrm{corr}}_{\mathrm{surf,vib}}$ for the individual systems. Note, that these are included in the reported adsorption energies in Supplementary Table S1.

Surface charging

All unknown quantities of the derived expressions in the harmonic CHE+DL approximation are properties at the PZC, which are parametrized using first-principles calculations, as discussed in the main text. This includes in particular the excess energy of the clean surface, the average adsorption energy as a function of coverage and the potentials of zero charge $\Phi_0^{\theta_\mathrm{a}}$; all determined under charge-neutral conditions. The yet undetermined interfacial capacitances at PZC $C_0^{\theta_\mathrm{a}}$, are determined with the described implicit solvent model and by finite surface charging (8 non-zero, net surface charges).

The net surface charges $n_e^{\mathrm{net},\alpha}$ are given by the number of electrons beyond charge neutrality used to charge the double layer. They are related to the absolute surface charges mentioned in the main text via

$$n_e^{\mathrm{abs},\alpha} = \theta_\mathrm{a}^\alpha \frac{q_\mathrm{a}}{e} + n_e^{\mathrm{net},\alpha} \ . \tag{1}$$

The differential interfacial capacitance for given configuration α can be obtained from the work function dependence on the net surface charges. As shown in previous works[S9] it is approximately independent on the potential, leading to the employed, harmonic CHE+DL

model of this work[S9] (cf. eq. 7 of the main text).

Therefore the interfacial capacitances C_0^α are obtained by polynomial interpolation of $\Phi^\alpha(n_e^{\mathrm{net}})$ – the work function dependence on the net surface charges – and taking the first order expansion term as estimated capacitance value (cf. Supplementary Fig. S5). The accuracy of the harmonic CHE+DL approximation[S9] and the good agreement with fully grand canonical implicit solvent calculations for the studied systems can be seen by the observed approximate linear and quadratic dependence on the net surface charges of the work function and the total energy, respectively (Supplementary Figs. S5 and S6).

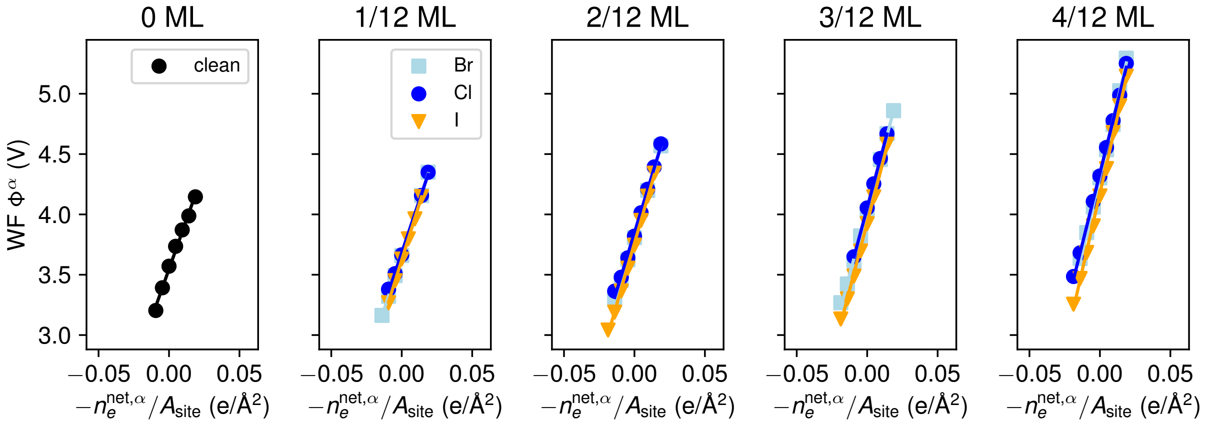

Figure S5: Work function Φ^α as a function of net surface charges $n_e^{\mathrm{net},\alpha}/A_{\mathrm{site}}$ for the considered systems, with quadratic fit.

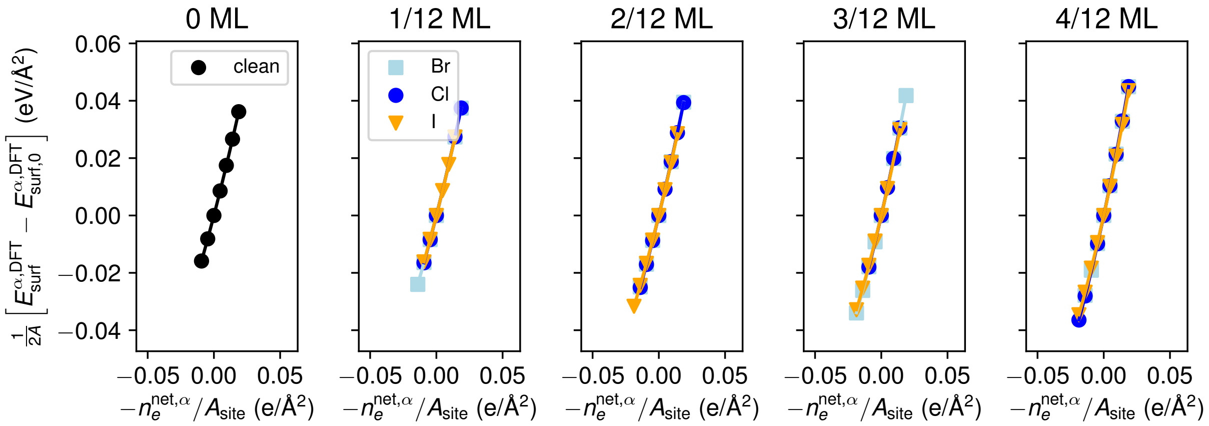

Figure S6: Total DFT energy as a function of net surface charges $n_e^{\mathrm{net},\alpha}/A_{\mathrm{site}}$ for the considered systems, with quadratic fit.

Simulated CVs using the revPBE functional

Coverage-dependent adsorption energies as well as PZCs were obtained with consistent computational parameters for the revPBE functional (based on in-solvent-relaxed revPBE structures at the revPBE lattice constant). This data in combination with the PBE-determined interfacial capacitances yields the theoretical revPBE CVs as plotted in Supplementary Fig. S7, which clearly agree less than the PBE results with the experimental CV curves.

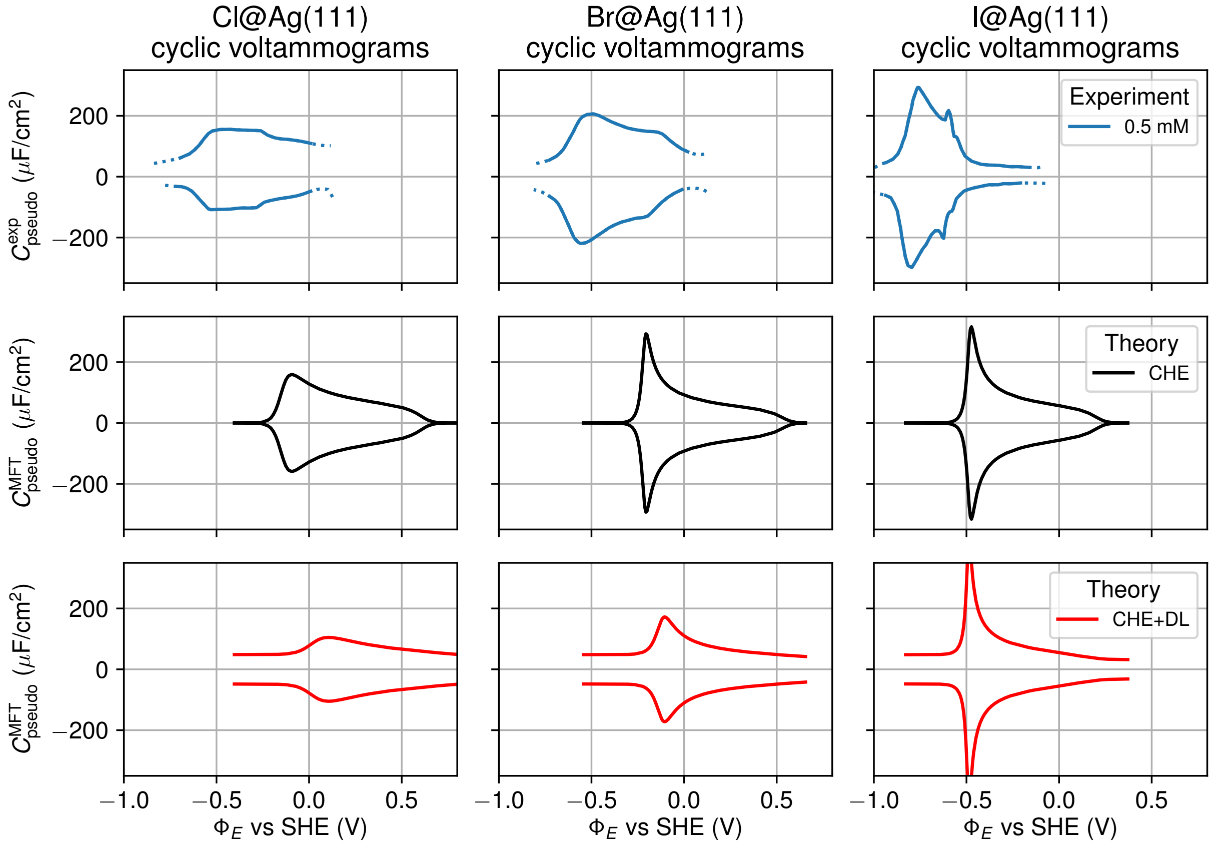

Figure S7: Top panels: Experimental cyclic voltammograms (CVs) by Foresti *et al.*[S1] in the potential range of halide electrosorption (c = 0.5 mM) on Ag(111). Middle panels: Corresponding simulated CVs for the CHE approach using the revPBE functional. Bottom panels: Simulated CVs for the CHE+DL approach using the revPBE functional.

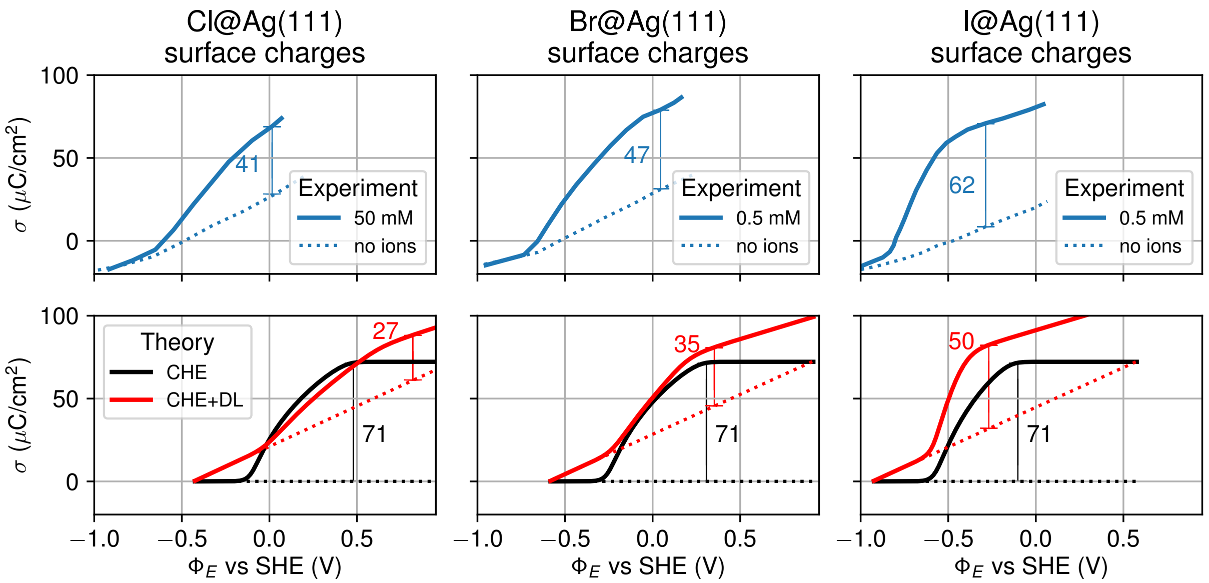

Figure S8: Surface charges for Ag(111) electrodes in ion-free (dotted lines) and ion-containing solutions (dashed lines) for the three considered system. Vertical bars indicate the charge difference between the adsorbate-covered and clean surfaces, determined at potentials of full coverage (see text). Top panels: Experimental results from Foresti *et al.*[S1]. Bottom panels: Corresponding theoretical results at the indicated experimental ion concentrations.

Simulated and experimental surface charges

We complement here the results of the main text by a plot of the simulated, equilibrium surface charges $\sigma(\Phi_E) = -en_e^{\mathrm{abs},\bar{\theta}_\mathrm{a}}/A_\mathrm{site}$ and the respective quantity measured in the experiments.

Fig. S8 plots σ for Ag(111) electrodes in ion-free (dotted lines) and ion-containing solutions (dashed lines) for the three considered system. The top panels report experimental results from Ref. S1; the bottom panels the respective theoretical results at corresponding ion concentrations. The CHE and CHE+DL curves in the figure refer to evaluating both, the equilibrium coverage as well as the expression for n_e^abs with the respective CHE terms only and adding also the DL terms (underlined terms in the main text), respectively. Note that surface charges are only defined up to a (arbitrary integration) constant, which is why a comparison of theory and experiment should only be made for the trends and charge differences within the plots.

In all plots we added as vertical bar the charge difference between the adsorbate-covered (solid lines) and clean surface (dotted lines). In agreement with the approach used in Ref. S1 to estimate the *integral electrosorption valencies* we determine this charge difference at a potential where the slope of clean and covered surfaces become essentially equal. In the theoretical curves we use the potential where the coverage is 99% the maximum coverage (1/3 ML). Again, the misalignment on the potential scale originates from the accuracy limitations of the predicted adsorption energies by first-principles methods, and will not further be analysed.

As DL charging is not captured within the CHE model, it is not surprising that the CHE results for the clean surface (black dotted lines) are dramatically different than the experimental results (blue dotted lines) and the CHE+DL (red dotted lines) model. The good agreement between experimental and CHE+DL slopes is testimony to the good interfacial capacitance values within the used implicit solvent model.

Turning to the ion containing solutions, we find nearly no ion-specificity in the CHE approach: all simulated charge vs potential curves exhibit nearly identical shapes. At variance, the theoretical CHE+DL and experimental curves exhibit an increasing slope going from Cl to I in regions of adsorption. At the same time the charge difference between clean and adsorbate-covered surfaces increases going form Cl to I. This increased slope in the CHE+DL calculations is due to two factors, 1. the narrowing of the potential range where adsorption happens (see main text) and 2. the increase in the absolute number of exchanged electrons per adsorbate as evidenced by the increased charge difference and measured by a variation in the electrosorption valency.

The integral electrosorption valency can be determined from these calculations using the identical method of Foresti *et al.*[S1], by dividing the indicated surface charge difference by the corresponding 1/3 ML coverage yielding theoretical values of -0.38(Cl), -0.49(Br) and -0.70(I) for the CHE+DL approach and -1 in all cases for the CHE method. The experimental values are -0.44(Cl), -0.65(Br), -0.86(I)[S1], thus underlining the fact that the CHE+DL results can

correctly reproduce the observed trend across the halide series and yield correct orders of magnitude, while the CHE method is inherently not able to make any such discrimination.

Note also that the total integrated charge in the CHE method (black vertical bars, 71 μC/cm^2) – which is directly related to the assumed maximum surface coverage of 1/3 ML – is significantly larger than the experimental values (41-62 μC/cm^2). This indicates that our setting of $\theta_{\mathrm{a}}^{\mathrm{max}} = 1/3$ ML can not be far off the experimental values. Indeed a naive (CHE-like) translation of surface charge into surface coverage indicates rather lower maximum surface coverages than those studied in our model.

Possible larger values for $\theta_{\mathrm{a}}^{\mathrm{max}}$ (that were not included in the present study) could naturally only lead to a further increase in the theoretical surface charge values. This would then lead to an even larger deviation of the CHE results from the experiments, while improving the CHE+DL predictions

This convinces us that an additional inclusion of higher coverages in the theoretical model could only make a stronger case for the CHE+DL method, which our present study did not include due to the strong experimental evidence that this is not needed, for the study of the lower-potential CV peak.

Tabulated Data

All numerical values for adsorption energies, work functions and the interfacial capacitance at PZC as obtained with the PBE functional in the implicit solvent setup are tabulated in table S1. Results for the revPBE functional are in table S2.

Table S1: Properties of halogen-covered and pristine Ag(111) surfaces as obtained by relaxations using the PBE functional in implicit solvent.

coverage	$\theta_a = 0$	$\theta_a = \frac{1}{12}$	$\theta_a = \frac{2}{12}$	$\theta_a = \frac{3}{12}$	$\theta_a = \frac{4}{12}$
Cl@Ag(111)					
$\bar{G}^{\theta_a}_{ads,0}$ (eV)	-	-1.571	-1.547	-1.500	-1.444
$\Phi^{\theta_a}_0$ (V)	3.57	3.66	3.82	4.05	4.32
$C^{\theta_a}_0$ (μF/cm^2)	48.3	46.1	41.8	36.8	34.0
Br@Ag(111)					
$\bar{G}^{\theta_a}_{ads,0}$ (eV)	-	-1.434	-1.411	-1.367	-1.312
$\Phi^{\theta_a}_0$ (V)	"	3.66	3.81	4.03	4.30
$C^{\theta_a}_0$ (μF/cm^2)	"	44.4	40.8	36.8	31.8
I@Ag(111)					
$\bar{G}^{\theta_a}_{ads,0}$ (eV)	-	-1.215	-1.196	-1.165	-1.122
$\Phi^{\theta_a}_0$ (V)	"	3.63	3.75	3.92	4.16
$C^{\theta_a}_0$ (μF/cm^2)	"	43.0	39.7	35.7	31.4

Table S2: Properties of halogen-covered and pristine Ag(111) surfaces as obtained by relaxations using the revPBE functional in implicit solvent.

coverage	$\theta_a = 0$	$\theta_a = \frac{1}{12}$	$\theta_a = \frac{2}{12}$	$\theta_a = \frac{3}{12}$	$\theta_a = \frac{4}{12}$
Cl@Ag(111)					
$\bar{G}^{\theta_a}_{ads,0}$ (eV)	-	-	-1.561	-1.497	-1.414
$\Phi^{\theta_a}_0$ (V)	3.39	-	3.71	3.92	4.19
Br@Ag(111)					
$\bar{G}^{\theta_a}_{ads,0}$ (eV)	-	-1.409	-1.386	-1.327	-1.242
$\Phi^{\theta_a}_0$ (V)	"	3.51	3.67	3.91	4.19
I@Ag(111)					
$\bar{G}^{\theta_a}_{ads,0}$ (eV)	-	-1.142	-1.117	-1.073	-0.990
$\Phi^{\theta_a}_0$ (V)	"	3.47	3.61	3.82	4.07

References

(S1) Foresti, M. L.; Innocenti, M.; Forni, F.; Guidelli, R. Electrosorption Valency and Partial Charge Transfer in Halide and Sulfide Adsorption on Ag(111). *Langmuir* **1998**, *14*, 7008–7016.

(S2) http://www.quantum-environment.org.

(S3) Andreussi, O.; Dabo, I.; Marzari, N. Revised self-consistent continuum solvation in electronic-structure calculations. *J. Chem. Phys.* **2012**, *136*, 064102.

(S4) Andreussi, O.; Marzari, N. Electrostatics of solvated systems in periodic boundary conditions. *Phys. Rev. B* **2014**, *90*, 245101.

(S5) Nattino, F.; Truscott, M.; Marzari, N.; Andreussi, O. Continuum models of the electrochemical diffuse layer in electronic-structure calculations. *J. Chem. Phys.* **2019**, *150*, 041722.

(S6) Hörmann, N. G.; Andreussi, O.; Marzari, N. Grand canonical simulations of electrochemical interfaces in implicit solvation models. *J. Chem. Phys.* **2019**, *150*, 041730.

(S7) Sundararaman, R.; Figueiredo, M. C.; Koper, M. T. M.; Schwarz, K. A. Electrochemical Capacitance of CO-Terminated Pt(111) Dominated by the CO–Solvent Gap. *J. Phys. Chem. Lett.* **2017**, *8*, 5344–5348.

(S8) Ringe, S.; Clark, E. L.; Resasco, J.; Walton, A.; Seger, B.; Bell, A. T.; Chan, K. Understanding cation effects in electrochemical CO2 reduction. *Energy Environ. Sci.* **2019**, *12*, 3001–3014.

(S9) Hörmann, N. G.; Marzari, N.; Reuter, K. Electrosorption at metal surfaces from first principles. *npj Comput. Mater.* **2020**, *6*, 136.

(S10) Harris, R. C.; Boschitsch, A. H.; Fenley, M. O. Sensitivities to parameterization in the size-modified Poisson-Boltzmann equation. *J. Chem. Phys.* **2014**, *140*, 075102.

(S11) Andreussi, O.; Nattino, F.; Hörmann, N. G. In *Atomic-Scale Modelling of Electrochemical Systems*; Melander, M., Laurila, T., Laasonen, K., Eds.; Copyright (IN PRESS) John Wiley and Sons Ltd. Please contact publishers for details, 2020.
